# Dichlorido{[2-(diphenyl­phosphino)phenyl­imino­meth­yl]ferrocene-κ^2^ *N*,*P*}platinum(II) dichloro­methane hemisolvate

**DOI:** 10.1107/S1600536808043493

**Published:** 2009-01-08

**Authors:** Yu Xie, Ya Wei, Jin-Gang Hu, Jin-Sheng Zhao, Feng-Xing Jiang

**Affiliations:** a Key Laboratory of Nondestructive Testing of the Ministry of Education, Nanchang Hangkong University, Nanchang 330063, People’s Republic of China; b Department of Chemistry, Liaocheng University, Liaocheng 252059, People’s Republic of China

## Abstract

In the title compound, [FePt(C_5_H_5_)(C_24_H_19_NP)Cl_2_]·0.5CH_2_Cl_2_, the Pt^II^ atom adopts a distorted square-planar geometry defined by one P atom and one N atom from the bidentate [2-(diphenyl­phosphino)phenyl­imino­meth­yl]ferro­cene ligand and two Cl atoms. Two disordered dichloro­methane solvent mol­ecules are each 0.25-occupied on a twofold rotation axis.

## Related literature

For general background, see: Cullen & Woolins (1981 ▶); Farrell *et al.* (2002 ▶); Gul *et al.* (2002 ▶); Wu *et al.* (2001 ▶). For the ligand synthesis, see: Gong *et al.* (2006 ▶); Zhang *et al.* (2006 ▶).
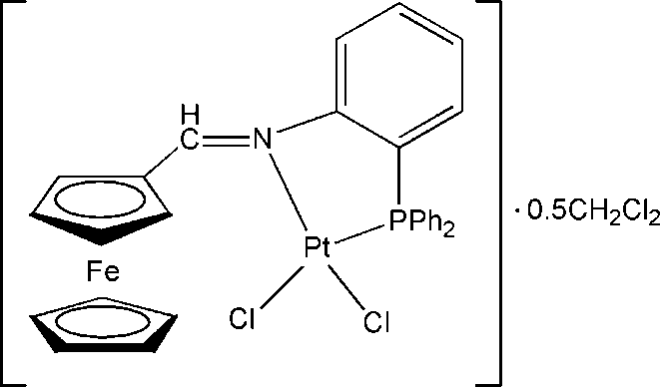


## Experimental

### 

#### Crystal data

[FePt(C_5_H_5_)(C_24_H_19_NP)Cl_2_]·0.5CH_2_Cl_2_
*M* *_r_* = 781.75
Orthorhombic, 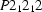

*a* = 19.5045 (11) Å
*b* = 12.0182 (7) Å
*c* = 12.9800 (8) Å
*V* = 3042.6 (3) Å^3^
*Z* = 4
Mo *K*α radiation
μ = 5.40 mm^−1^
*T* = 293 (2) K
0.3 × 0.1 × 0.1 mm

#### Data collection

Siemens SMART CCD diffractometer
Absorption correction: multi-scan (*SADABS*; Sheldrick, 1996 ▶) *T*_min_ = 0.532, *T*_max_ = 0.588
19964 measured reflections
5902 independent reflections
4954 reflections with *I* > 2σ(*I*)
*R*_int_ = 0.058

#### Refinement

*R*[*F* ^2^ > 2σ(*F* ^2^)] = 0.043
*wR*(*F* ^2^) = 0.116
*S* = 1.04
5902 reflections
345 parameters
24 restraints
H-atom parameters constrained
Δρ_max_ = 1.18 e Å^−3^
Δρ_min_ = −0.93 e Å^−3^
Absolute structure: Flack (1983 ▶), 856 Friedel pairs
Flack parameter: 0.002 (11)

### 

Data collection: *SMART* (Siemens, 1996 ▶); cell refinement: *SAINT* (Siemens, 1996 ▶); data reduction: *SAINT*; program(s) used to solve structure: *SHELXS97* (Sheldrick, 2008 ▶); program(s) used to refine structure: *SHELXL97* (Sheldrick, 2008 ▶); molecular graphics: *SHELXTL* (Sheldrick, 2008 ▶); software used to prepare material for publication: *SHELXTL*.

## Supplementary Material

Crystal structure: contains datablocks global, I. DOI: 10.1107/S1600536808043493/hy2171sup1.cif

Structure factors: contains datablocks I. DOI: 10.1107/S1600536808043493/hy2171Isup2.hkl

Additional supplementary materials:  crystallographic information; 3D view; checkCIF report

## Figures and Tables

**Table 1:** Selected bond lengths (Å)

Pt—N	2.031 (3)
Pt—P	2.2089 (10)
Pt—Cl1	2.2996 (13)
Pt—Cl2	2.3673 (11)

**Fig. 1.**
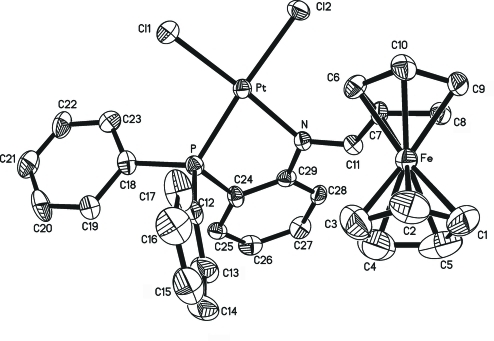
The molecular structure of the title compound. Displacement ellipsoids are drawn at the 30% probability level. H atoms and disordered dichloromethane solvent molecules have been omitted for clarity.

## supplementary crystallographic information

### Comment

Ferrocene derivatives containing heteroatoms with good donor abilities have attracted great interest during the last few years in organometallic chemistry, since the coordination of a metal to these heteroatoms produces polymetallic molecules (Cullen & Woolins, 1981). Some examples showing the utility of these polymetallic compounds in homogeneous catalysis (Farrell *et al.*, 2002; Wu *et al.*, 2001) or for the design of new materials with outstanding properties (Gul *et al.*, 2002) have been described. Here we report a new bimetallic platinum(II) complex with a ferrocenyliminophosphine ligand.

In the title compound, the Pt^II^ atom adopts a distorted square planar geometry, defined by one P atom and one N atom from the bidentate ferrocenyliminophosphine ligand (*L*) and two Cl atoms (Fig. 1), with N—Pt—Cl1 = 176.29 (9)° and P—Pt—Cl2 = 170.19 (4)°. The difference between the Pt—Cl bond lengths [2.2996 (13) and 2.3673 (11) Å] (Table 1) reflects the stronger *trans* influence of the tertiary phosphine compared with the imino group. The ligand *L* adopts a five-membered chelating ring. The benzene ring and the cyclopentadienyl ring are *trans*, with respect to the C11—N double bond.

### Experimental

The ligand *L* was prepared by literature method (Gong *et al.*, 2006; Zhang *et al.*, 2006). The title compound was prepared by reacting equal molar K_2_PtCl_4_ and *L* in CH_2_Cl_2_ (yield 83%). Brown needle crystals suitable for X-ray analysis were obtained by vapor diffusion of diethyl ether into a solution of the title compound in CH_2_Cl_2_. Analysis calculated for C_29.5_H_25_Cl_3_FeNPPt: C 45.32, H 3.22, N 1.79%; found: C 45.61, H 3.33, N 1.70%.

### Refinement

H atoms were positioned geomertrically and treated as riding, with C—H = 0.98 (cyclopentadienyl), 0.93 (phenyl) and 0.96 (CH_2_) Å and with *U*_iso_(H) = 1.2*U*_eq_(C). The highest residual electron density was found 0.67 Å from atom Cl4 and the deepest hole 0.79 Å from atom Pt. The disordered dichloromethane solvent molecules are each 0.25-occupied on a twofold rotation axis.

### Crystal data

**Table:**

[FePt(C_5_H_5_)(C_24_H_19_NP)Cl_2_]·0.5CH_2_Cl_2_	*F*(000) = 1516
*M**_r_* = 781.75	*D*_x_ = 1.707 Mg m^−^^3^
Orthorhombic, *P*2_1_2_1_2	Mo *K*α radiation, λ = 0.71073 Å
Hall symbol: P 2 2ab	Cell parameters from 892 reflections
*a* = 19.5045 (11) Å	θ = 3.2–25.8°
*b* = 12.0182 (7) Å	µ = 5.40 mm^−^^1^
*c* = 12.9800 (8) Å	*T* = 293 K
*V* = 3042.6 (3) Å^3^	Needle, brown–yellow
*Z* = 4	0.3 × 0.1 × 0.1 mm

### Data collection

**Table:**

Siemens SMART CCD diffractometer	5902 independent reflections
Radiation source: fine-focus sealed tube	4954 reflections with *I* > 2σ(*I*)
graphite	*R*_int_ = 0.058
ω scans	θ_max_ = 26.0°, θ_min_ = 1.9°
Absorption correction: multi-scan (*SADABS*; Sheldrick, 1996)	*h* = −23→24
*T*_min_ = 0.532, *T*_max_ = 0.588	*k* = −14→14
19964 measured reflections	*l* = −16→15

### Refinement

**Table:**

Refinement on *F*^2^	Secondary atom site location: difference Fourier map
Least-squares matrix: full	Hydrogen site location: inferred from neighbouring sites
*R*[*F*^2^ > 2σ(*F*^2^)] = 0.043	H-atom parameters constrained
*wR*(*F*^2^) = 0.116	*w* = 1/[σ^2^(*F*_o_^2^) + (0.05*P*)^2^] where *P* = (*F*_o_^2^ + 2*F*_c_^2^)/3
*S* = 1.04	(Δ/σ)_max_ = 0.024
5902 reflections	Δρ_max_ = 1.18 e Å^−^^3^
345 parameters	Δρ_min_ = −0.93 e Å^−^^3^
24 restraints	Absolute structure: Flack (1983), 856 Friedel pairs
Primary atom site location: structure-invariant direct methods	Flack parameter: 0.002 (11)

### Fractional atomic coordinates and isotropic or equivalent isotropic displacement parameters (Å^2^)

**Table:**

	*x*	*y*	*z*	*U*_iso_*/*U*_eq_	Occ. (<1)
Pt	0.270357 (7)	0.911967 (14)	0.143789 (12)	0.03558 (4)	
Fe	0.34183 (3)	0.52770 (6)	0.19921 (5)	0.04447 (19)	
Cl1	0.32009 (6)	1.06905 (11)	0.21198 (10)	0.0568 (4)	
Cl2	0.35362 (5)	0.90898 (12)	0.01112 (9)	0.0538 (3)	
Cl3	0.4874 (2)	0.8826 (5)	0.3249 (4)	0.167 (2)	0.50
Cl4	0.06774 (11)	0.54821 (19)	0.23620 (19)	0.0506 (7)	0.50
P	0.17978 (5)	0.91268 (11)	0.24594 (8)	0.0378 (3)	
N	0.22282 (15)	0.7723 (3)	0.0921 (2)	0.0337 (9)	
C1	0.3477 (4)	0.3728 (6)	0.2664 (5)	0.097 (3)	
H1A	0.3771	0.3110	0.2447	0.117*	
C2	0.3629 (4)	0.4509 (6)	0.3371 (4)	0.096 (3)	
H2A	0.4066	0.4563	0.3742	0.115*	
C3	0.3087 (4)	0.5257 (7)	0.3468 (5)	0.102 (2)	
H3A	0.3067	0.5896	0.3936	0.123*	
C4	0.2585 (4)	0.4921 (8)	0.2825 (6)	0.112 (2)	
H4A	0.2137	0.5282	0.2749	0.135*	
C5	0.2797 (4)	0.3974 (6)	0.2289 (6)	0.110 (3)	
H5A	0.2530	0.3543	0.1789	0.132*	
C6	0.3711 (2)	0.6848 (4)	0.1581 (3)	0.0451 (13)	
H6A	0.3666	0.7517	0.2007	0.054*	
C7	0.3221 (2)	0.6444 (4)	0.0886 (3)	0.0402 (12)	
C8	0.3478 (2)	0.5451 (4)	0.0462 (3)	0.0442 (13)	
H8A	0.3246	0.4984	−0.0050	0.053*	
C9	0.4145 (2)	0.5275 (4)	0.0860 (4)	0.0482 (13)	
H9A	0.4452	0.4658	0.0686	0.058*	
C10	0.4278 (2)	0.6129 (4)	0.1561 (4)	0.0539 (15)	
H10A	0.4696	0.6210	0.1973	0.065*	
C11	0.25027 (18)	0.6757 (4)	0.0722 (3)	0.0360 (11)	
H11A	0.2215	0.6215	0.0449	0.043*	
C12	0.1890 (2)	0.8272 (4)	0.3601 (4)	0.0516 (13)	
C13	0.1388 (3)	0.7539 (5)	0.3915 (4)	0.0752 (19)	
H13A	0.0985	0.7462	0.3539	0.090*	
C14	0.1495 (4)	0.6915 (6)	0.4806 (4)	0.090 (2)	
H14A	0.1174	0.6392	0.5022	0.108*	
C15	0.2068 (3)	0.7088 (6)	0.5338 (5)	0.092 (2)	
H15A	0.2139	0.6660	0.5925	0.111*	
C16	0.2540 (3)	0.7823 (6)	0.5090 (5)	0.0902 (19)	
H16A	0.2923	0.7946	0.5502	0.108*	
C17	0.2437 (3)	0.8398 (6)	0.4191 (4)	0.086 (2)	
H17A	0.2770	0.8904	0.3985	0.104*	
C18	0.1426 (2)	1.0435 (4)	0.2854 (3)	0.0443 (13)	
C19	0.1014 (3)	1.0460 (5)	0.3785 (4)	0.0586 (16)	
H19A	0.0966	0.9831	0.4195	0.070*	
C20	0.0699 (3)	1.1442 (5)	0.4040 (5)	0.0793 (19)	
H20A	0.0416	1.1462	0.4617	0.095*	
C21	0.0784 (3)	1.2379 (4)	0.3481 (5)	0.0659 (17)	
H21A	0.0579	1.3039	0.3693	0.079*	
C22	0.1165 (3)	1.2358 (5)	0.2618 (5)	0.0641 (17)	
H22A	0.1198	1.2999	0.2219	0.077*	
C23	0.1507 (2)	1.1413 (4)	0.2307 (4)	0.0505 (14)	
H23A	0.1792	1.1434	0.1732	0.061*	
C24	0.1200 (2)	0.8442 (4)	0.1607 (3)	0.0378 (11)	
C25	0.0481 (2)	0.8598 (4)	0.1615 (3)	0.0404 (12)	
H25A	0.0273	0.9038	0.2115	0.049*	
C26	0.0092 (2)	0.8074 (4)	0.0854 (4)	0.0536 (15)	
H26A	−0.0381	0.8164	0.0841	0.064*	
C27	0.0420 (2)	0.7399 (4)	0.0095 (3)	0.0506 (14)	
H27A	0.0159	0.7044	−0.0406	0.061*	
C28	0.1120 (2)	0.7269 (4)	0.0100 (3)	0.0410 (12)	
H28A	0.1336	0.6825	−0.0389	0.049*	
C29	0.14973 (19)	0.7811 (3)	0.0845 (3)	0.0372 (11)	
C30	0.5000	1.0000	0.2754 (18)	0.104 (8)	0.50
H30A	0.5386	0.9885	0.2308	0.125*	0.50
C31	0.0000	0.5000	0.1697 (9)	0.051 (4)	0.50
H31A	−0.0159	0.5590	0.1260	0.061*	0.50

### Atomic displacement parameters (Å^2^)

**Table:**

	*U*^11^	*U*^22^	*U*^33^	*U*^12^	*U*^13^	*U*^23^
Pt	0.03605 (6)	0.03424 (7)	0.03646 (7)	0.00216 (7)	−0.00032 (7)	0.00005 (8)
Fe	0.0432 (3)	0.0405 (4)	0.0497 (4)	0.0021 (3)	0.0018 (3)	−0.0005 (3)
Cl1	0.0584 (6)	0.0514 (7)	0.0608 (7)	−0.0109 (6)	0.0017 (5)	−0.0103 (6)
Cl2	0.0508 (5)	0.0506 (6)	0.0598 (6)	0.0014 (6)	0.0200 (5)	0.0055 (6)
Cl3	0.131 (3)	0.146 (5)	0.223 (5)	−0.030 (3)	−0.087 (3)	0.041 (4)
Cl4	0.0373 (10)	0.0439 (13)	0.0706 (14)	−0.0113 (9)	−0.0071 (10)	−0.0009 (11)
P	0.0425 (5)	0.0380 (6)	0.0329 (5)	0.0046 (6)	0.0017 (4)	0.0009 (5)
N	0.0341 (15)	0.0340 (17)	0.0331 (15)	0.0043 (15)	0.0016 (14)	−0.0008 (14)
C1	0.132 (5)	0.060 (4)	0.100 (5)	−0.006 (4)	0.029 (4)	0.016 (3)
C2	0.119 (5)	0.115 (6)	0.054 (3)	−0.001 (4)	0.009 (3)	0.029 (3)
C3	0.124 (4)	0.104 (4)	0.078 (3)	0.008 (3)	0.032 (3)	−0.005 (3)
C4	0.097 (4)	0.117 (4)	0.123 (4)	0.006 (3)	0.030 (3)	0.021 (4)
C5	0.123 (5)	0.113 (5)	0.095 (5)	−0.060 (5)	−0.009 (4)	0.023 (4)
C6	0.046 (2)	0.038 (2)	0.051 (3)	−0.004 (2)	−0.010 (2)	−0.004 (2)
C7	0.051 (2)	0.031 (2)	0.039 (2)	0.009 (2)	0.001 (2)	−0.0032 (19)
C8	0.038 (2)	0.045 (3)	0.049 (2)	0.004 (2)	−0.0016 (19)	−0.003 (2)
C9	0.036 (2)	0.042 (3)	0.066 (3)	0.005 (2)	0.002 (2)	−0.006 (2)
C10	0.0300 (18)	0.065 (3)	0.067 (3)	0.004 (2)	−0.006 (2)	−0.011 (3)
C11	0.0337 (19)	0.044 (2)	0.0302 (19)	0.0036 (17)	−0.0006 (16)	0.0032 (19)
C12	0.063 (2)	0.047 (3)	0.044 (2)	0.020 (2)	0.016 (2)	−0.005 (2)
C13	0.095 (4)	0.068 (4)	0.063 (3)	0.015 (3)	0.018 (3)	0.018 (3)
C14	0.129 (5)	0.075 (4)	0.067 (3)	0.004 (4)	0.045 (3)	0.023 (3)
C15	0.121 (4)	0.087 (4)	0.069 (3)	0.035 (3)	0.006 (3)	0.011 (3)
C16	0.093 (3)	0.095 (4)	0.083 (3)	0.028 (3)	−0.014 (3)	0.018 (3)
C17	0.108 (4)	0.114 (5)	0.037 (2)	0.045 (4)	−0.034 (3)	−0.007 (3)
C18	0.040 (2)	0.049 (3)	0.044 (2)	0.013 (2)	−0.0078 (19)	−0.007 (2)
C19	0.065 (3)	0.058 (3)	0.053 (3)	−0.002 (3)	0.011 (2)	−0.011 (2)
C20	0.070 (3)	0.093 (4)	0.075 (3)	0.038 (3)	0.015 (3)	−0.029 (3)
C21	0.071 (3)	0.037 (3)	0.090 (4)	0.016 (2)	−0.010 (3)	−0.006 (3)
C22	0.064 (3)	0.042 (3)	0.085 (4)	0.010 (3)	−0.007 (3)	−0.017 (3)
C23	0.052 (2)	0.048 (3)	0.051 (3)	−0.003 (2)	−0.007 (2)	0.002 (2)
C24	0.048 (2)	0.039 (2)	0.026 (2)	0.0119 (19)	0.0011 (18)	0.0000 (18)
C25	0.0375 (19)	0.042 (2)	0.042 (2)	−0.0011 (19)	0.0028 (18)	0.003 (2)
C26	0.039 (2)	0.056 (3)	0.065 (3)	0.005 (2)	−0.004 (2)	0.017 (3)
C27	0.047 (2)	0.051 (3)	0.054 (3)	0.003 (2)	−0.018 (2)	0.001 (2)
C28	0.040 (2)	0.028 (2)	0.055 (3)	0.0042 (19)	0.003 (2)	−0.0047 (19)
C29	0.0346 (18)	0.029 (2)	0.048 (2)	0.0037 (18)	0.0009 (18)	0.0088 (19)
C30	0.017 (6)	0.121 (17)	0.174 (19)	0.001 (8)	0.000	0.000
C31	0.093 (9)	0.020 (6)	0.040 (7)	−0.019 (7)	0.000	0.000

### Geometric parameters (Å, °)

**Table:**

Pt—N	2.031 (3)	C9—H9A	0.9800
Pt—P	2.2089 (10)	C10—H10A	0.9800
Pt—Cl1	2.2996 (13)	C11—H11A	0.9300
Pt—Cl2	2.3673 (11)	C12—C17	1.323 (7)
Fe—C1	2.059 (7)	C12—C13	1.379 (8)
Fe—C2	2.055 (6)	C13—C14	1.394 (8)
Fe—C3	2.022 (7)	C13—H13A	0.9300
Fe—C4	1.998 (8)	C14—C15	1.330 (9)
Fe—C5	2.017 (7)	C14—H14A	0.9300
Fe—C6	2.043 (5)	C15—C16	1.317 (10)
Fe—C7	2.044 (4)	C15—H15A	0.9300
Fe—C8	2.000 (5)	C16—C17	1.371 (9)
Fe—C9	2.041 (5)	C16—H16A	0.9300
Fe—C10	2.042 (5)	C17—H17A	0.9300
Cl3—C30	1.570 (11)	C18—C23	1.382 (7)
Cl4—C31	1.681 (6)	C18—C19	1.451 (6)
P—C18	1.806 (5)	C19—C20	1.371 (8)
P—C24	1.806 (4)	C19—H19A	0.9300
P—C12	1.812 (5)	C20—C21	1.350 (8)
N—C11	1.304 (5)	C20—H20A	0.9300
N—C29	1.433 (5)	C21—C22	1.345 (8)
C1—C2	1.346 (10)	C21—H21A	0.9300
C1—C5	1.443 (10)	C22—C23	1.378 (7)
C1—H1A	0.9800	C22—H22A	0.9300
C2—C3	1.393 (10)	C23—H23A	0.9300
C2—H2A	0.9800	C24—C29	1.375 (6)
C3—C4	1.348 (11)	C24—C25	1.413 (6)
C3—H3A	0.9800	C25—C26	1.397 (6)
C4—C5	1.396 (11)	C25—H25A	0.9300
C4—H4A	0.9800	C26—C27	1.427 (7)
C5—H5A	0.9800	C26—H26A	0.9300
C6—C7	1.401 (6)	C27—C28	1.375 (6)
C6—C10	1.404 (6)	C27—H27A	0.9300
C6—H6A	0.9800	C28—C29	1.378 (6)
C7—C8	1.407 (6)	C28—H28A	0.9300
C7—C11	1.466 (6)	C30—Cl3^i^	1.570 (11)
C8—C9	1.415 (6)	C30—H30A	0.9600
C8—H8A	0.9800	C31—Cl4^ii^	1.681 (6)
C9—C10	1.396 (7)	C31—H31A	0.9600
			
N—Pt—P	80.59 (9)	C10—C6—Fe	69.9 (3)
N—Pt—Cl1	176.29 (9)	C7—C6—H6A	125.9
P—Pt—Cl1	95.92 (4)	C10—C6—H6A	125.9
N—Pt—Cl2	93.46 (9)	Fe—C6—H6A	125.9
P—Pt—Cl2	170.19 (4)	C6—C7—C8	107.6 (4)
Cl1—Pt—Cl2	90.17 (4)	C6—C7—C11	131.0 (4)
C4—Fe—C8	127.4 (3)	C8—C7—C11	120.1 (4)
C4—Fe—C5	40.7 (3)	C6—C7—Fe	69.9 (3)
C8—Fe—C5	107.8 (3)	C8—C7—Fe	68.0 (3)
C4—Fe—C3	39.2 (3)	C11—C7—Fe	117.3 (3)
C8—Fe—C3	163.9 (2)	C7—C8—C9	108.2 (4)
C5—Fe—C3	67.5 (3)	C7—C8—Fe	71.3 (3)
C4—Fe—C9	162.5 (3)	C9—C8—Fe	71.1 (3)
C8—Fe—C9	40.96 (18)	C7—C8—H8A	125.9
C5—Fe—C9	123.6 (3)	C9—C8—H8A	125.9
C3—Fe—C9	154.7 (2)	Fe—C8—H8A	125.9
C4—Fe—C10	157.4 (3)	C10—C9—C8	107.4 (4)
C8—Fe—C10	68.14 (19)	C10—C9—Fe	70.1 (3)
C5—Fe—C10	159.2 (3)	C8—C9—Fe	68.0 (3)
C3—Fe—C10	121.9 (3)	C10—C9—H9A	126.3
C9—Fe—C10	39.98 (19)	C8—C9—H9A	126.3
C4—Fe—C6	124.5 (3)	Fe—C9—H9A	126.3
C8—Fe—C6	68.16 (18)	C9—C10—C6	108.6 (4)
C5—Fe—C6	159.2 (2)	C9—C10—Fe	70.0 (3)
C3—Fe—C6	110.3 (3)	C6—C10—Fe	69.9 (3)
C9—Fe—C6	67.63 (19)	C9—C10—H10A	125.7
C10—Fe—C6	40.19 (18)	C6—C10—H10A	125.7
C4—Fe—C7	111.9 (3)	Fe—C10—H10A	125.7
C8—Fe—C7	40.71 (18)	N—C11—C7	126.3 (4)
C5—Fe—C7	123.6 (2)	N—C11—H11A	116.9
C3—Fe—C7	127.8 (3)	C7—C11—H11A	116.9
C9—Fe—C7	68.04 (18)	C17—C12—C13	118.3 (5)
C10—Fe—C7	67.55 (18)	C17—C12—P	119.3 (4)
C6—Fe—C7	40.09 (17)	C13—C12—P	122.3 (4)
C4—Fe—C2	66.2 (3)	C12—C13—C14	118.9 (6)
C8—Fe—C2	154.1 (2)	C12—C13—H13A	120.6
C5—Fe—C2	66.8 (3)	C14—C13—H13A	120.6
C3—Fe—C2	40.0 (3)	C15—C14—C13	118.2 (6)
C9—Fe—C2	119.2 (2)	C15—C14—H14A	120.9
C10—Fe—C2	107.4 (3)	C13—C14—H14A	120.9
C6—Fe—C2	125.9 (2)	C16—C15—C14	124.5 (7)
C7—Fe—C2	163.2 (2)	C16—C15—H15A	117.8
C4—Fe—C1	67.8 (3)	C14—C15—H15A	117.8
C8—Fe—C1	120.8 (2)	C15—C16—C17	116.3 (6)
C5—Fe—C1	41.5 (3)	C15—C16—H16A	121.8
C3—Fe—C1	66.8 (3)	C17—C16—H16A	121.8
C9—Fe—C1	105.4 (2)	C12—C17—C16	123.7 (6)
C10—Fe—C1	121.6 (3)	C12—C17—H17A	118.2
C6—Fe—C1	158.5 (2)	C16—C17—H17A	118.2
C7—Fe—C1	158.2 (2)	C23—C18—C19	118.3 (4)
C2—Fe—C1	38.2 (3)	C23—C18—P	123.3 (3)
C18—P—C24	108.1 (2)	C19—C18—P	118.4 (4)
C18—P—C12	107.6 (2)	C20—C19—C18	117.9 (5)
C24—P—C12	107.9 (2)	C20—C19—H19A	121.1
C18—P—Pt	119.65 (15)	C18—C19—H19A	121.1
C24—P—Pt	98.46 (13)	C21—C20—C19	122.2 (5)
C12—P—Pt	114.18 (15)	C21—C20—H20A	118.9
C11—N—C29	117.4 (3)	C19—C20—H20A	118.9
C11—N—Pt	127.8 (3)	C22—C21—C20	120.0 (5)
C29—N—Pt	114.6 (2)	C22—C21—H21A	120.0
C2—C1—C5	106.8 (7)	C20—C21—H21A	120.0
C2—C1—Fe	70.7 (4)	C21—C22—C23	121.8 (5)
C5—C1—Fe	67.7 (4)	C21—C22—H22A	119.1
C2—C1—H1A	126.6	C23—C22—H22A	119.1
C5—C1—H1A	126.6	C22—C23—C18	119.6 (5)
Fe—C1—H1A	126.6	C22—C23—H23A	120.2
C1—C2—C3	110.1 (7)	C18—C23—H23A	120.2
C1—C2—Fe	71.1 (4)	C29—C24—C25	119.8 (4)
C3—C2—Fe	68.7 (4)	C29—C24—P	114.8 (3)
C1—C2—H2A	124.9	C25—C24—P	125.1 (3)
C3—C2—H2A	124.9	C26—C25—C24	118.3 (4)
Fe—C2—H2A	124.9	C26—C25—H25A	120.9
C4—C3—C2	107.6 (7)	C24—C25—H25A	120.9
C4—C3—Fe	69.5 (4)	C25—C26—C27	120.1 (4)
C2—C3—Fe	71.3 (4)	C25—C26—H26A	120.0
C4—C3—H3A	126.2	C27—C26—H26A	120.0
C2—C3—H3A	126.2	C28—C27—C26	120.4 (4)
Fe—C3—H3A	126.2	C28—C27—H27A	119.8
C3—C4—C5	109.7 (7)	C26—C27—H27A	119.8
C3—C4—Fe	71.3 (5)	C27—C28—C29	118.7 (4)
C5—C4—Fe	70.4 (4)	C27—C28—H28A	120.7
C3—C4—H4A	125.1	C29—C28—H28A	120.7
C5—C4—H4A	125.1	C24—C29—C28	122.7 (4)
Fe—C4—H4A	125.1	C24—C29—N	114.3 (4)
C4—C5—C1	105.7 (7)	C28—C29—N	123.0 (4)
C4—C5—Fe	68.9 (5)	Cl3^i^—C30—Cl3	131.7 (16)
C1—C5—Fe	70.8 (4)	Cl3^i^—C30—H30A	104.6
C4—C5—H5A	127.1	Cl3—C30—H30A	104.0
C1—C5—H5A	127.1	Cl4—C31—Cl4^ii^	118.2 (7)
Fe—C5—H5A	127.1	Cl4—C31—H31A	107.6
C7—C6—C10	108.2 (4)	Cl4^ii^—C31—H31A	107.7
C7—C6—Fe	70.0 (3)		

Symmetry codes: (i) −*x*+1, −*y*+2, *z*; (ii) −*x*, −*y*+1, *z*.
